# National survey of urologists' views on prostate cancer screening in the United Kingdom

**DOI:** 10.1002/bco2.70228

**Published:** 2026-05-17

**Authors:** Nikhil Mayor, Francesca Rawlins, Emma Cullen, Varsha Sonigra‐Patel, Rhian Gabe, Hashim U. Ahmed

**Affiliations:** ^1^ Imperial Prostate, Department of Surgery & Cancer, Imperial College London Hammersmith Hospital Campus London UK; ^2^ Urology Department, Imperial College Healthcare NHS Charing Cross Hospital London UK; ^3^ Centre for Evaluation and Methods, Barts Clinical Trials Unit, Wolfson Institute of Population Health Queen Mary University of London London UK

**Keywords:** National Screening Committee, prostate cancer, screening, survey, urologist

## 1 INTRODUCTION

Over the past two decades, the UK National Screening Committee (NSC) has reviewed the evidence for prostate cancer screening at regular intervals and consistently arrived at the conclusion that the harms of a screening programme based on prostate‐specific antigen (PSA) testing are likely to outweigh the benefits. The NSC's remit has now been expanded to allow review of evidence for targeted screening of high‐risk groups in addition to whole population screening. In late 2025, after an updated evidence review, the committee issued the following recommendations:

- **Do not offer** *whole population* prostate cancer screening.
- **Do not offer** *targeted* screening to men of Black ethnicity.
- **Do not offer** *targeted* screening to men with any first‐degree relative with breast, ovarian or prostate cancer.
- **Offer** *targeted* screening to men with a confirmed BRCA1/2 gene mutation every 2 years from the ages of 45 to 61.

The recommendations were opened to public and professional consultation before a final decision was made in spring of 2026. Such consultations generally garner substantial public response as well as lobbying from patient advocacy groups, charities and politicians, but the views of clinicians directly involved in diagnosing and treating prostate cancer are not routinely captured in this process. We therefore conducted a cross‐sectional, anonymised survey of urologists in England to capture professional perspectives on the draft recommendations.

A near‐complete list of practising urologists was compiled using publicly available sources and national directories. Survey invitations were distributed using the Qualtrics platform, with three reminder emails sent to non‐responders. Each urologist received a unique link associated with their email address, preventing duplicate submissions and restricting access to invited participants. The survey was not publicly accessible. Responses were collected anonymously, with no identifiable data or IP addresses recorded. Respondents were asked whether they agreed with each NSC recommendation and to rate the perceived strength of the supporting evidence using a 5‐point Likert scale. Demographic and professional characteristics were also collected with an optional free‐text question for additional comments. Results were analysed descriptively. Free‐text responses were grouped into themes.

**FIGURE 1:**
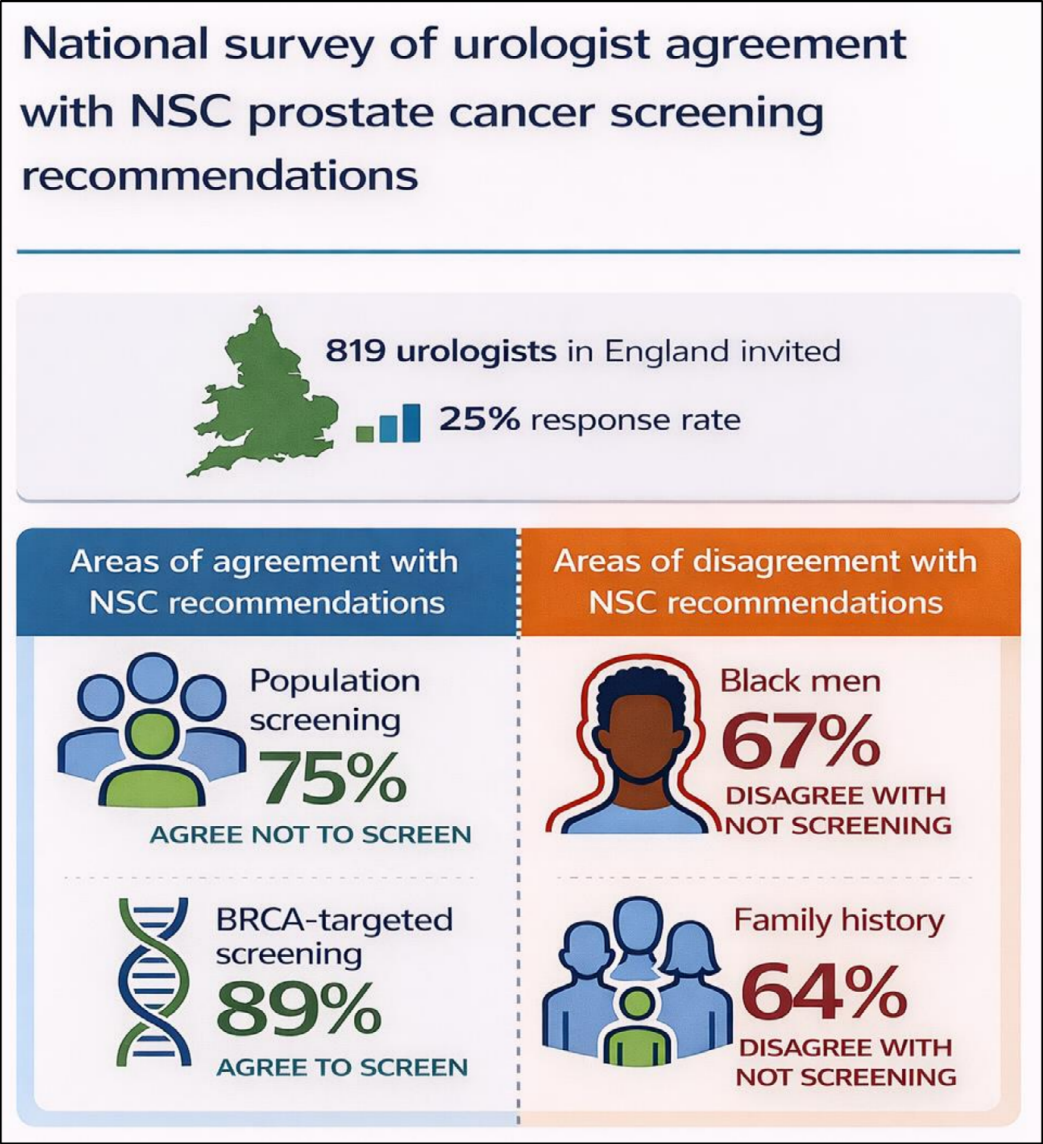
Open in figure viewer Infographic summarising survey results.

A total of 204 responses were received from 819 invited urologists (response rate of 24.9%), with representation from all seven NHS regions in England (Figure 1). Most respondents reported direct involvement in prostate cancer care (90% involved in diagnosis; 73% in treatment). Most respondents agreed with the recommendation not to introduce whole population prostate cancer screening (75%; 153/204), with the majority rating the supporting evidence as moderate or strong (83%; 166/202). There was very strong agreement with the recommendation to offer targeted screening to men with confirmed BRCA1/2 mutations (89%; 169/190), with 65% (125/191) rating the supporting evidence as strong or very strong. However, there was a clear divergence in responses to recommendations relating to targeted screening for Black men and those with a family history, with most disagreeing with the NSC recommendations. Only 33% (63/191) agreed with the recommendation not to screen Black men, and 36% (68/191) agreed with the recommendation not to screen men with any family history. Respondents reported low confidence in the supporting evidence, with 41% (79/191) and 43% (82/191) rating the evidence as weak or very weak for these two higher‐risk subgroups, respectively.

Free‐text responses highlighted several themes. There was recognition of the limitations of using PSA to screen the whole population, particularly due to concerns around overdiagnosis and overtreatment. In contrast, many expressed strong support for targeted screening in higher‐risk groups, particularly Black men, citing higher incidence and poorer outcomes. However, several respondents also noted that men of Black ethnicity have been historically under‐represented in large prostate cancer screening trials. Responses frequently highlighted that much of the evidence underpinning the recommendations reflects historical cohorts predating contemporary diagnostic pathways and emphasised the need for updated data. Many responses advocated for improved diagnostic tools and more refined risk‐stratified approaches using modern biomarkers and imaging. Practical challenges of implementing targeted screening were frequently highlighted, including difficulties in identifying men with family history or BRCA mutations and concerns regarding NHS capacity. Responses also noted that opportunistic PSA testing is already occurring in an unstructured and potentially inequitable manner.

Our survey provides a valuable insight into the views of UK urologists. There was broad agreement with recommendations not to offer screening at a whole population level. The majority of evidence for prostate cancer screening with long‐term cancer‐specific outcomes comes from the large screening trials of the 1990s and early 2000s where PSA followed by systematic biopsy was the prevailing pathway.^1^, ^2^, ^3^ Only the European Randomized Study of Screening for Prostate Cancer (ERSPC) showed a meaningful mortality benefit by using repeated PSA tests, but this was associated with unacceptable levels of overdiagnosis.^1^ Contemporary screening protocols including pre‐biopsy MRI and targeted‐only biopsy are likely to reduce the harms of screening, but cancer‐specific mortality data are still lacking.^4^ Respondents to the survey predominantly agreed that evidence for screening the whole population was weak.

There was strong agreement with the recommendation to offer targeted screening to men with confirmed BRCA1/2 mutations. The NSC modelling suggests that screening in this group would lead to less overdiagnosis compared with population screening.^5^ However, the model did not consider the cost of BRCA gene testing, whether screening is better targeted at one or both BRCA genes, or whether widespread genetic testing for BRCA1/2 is cost‐effective. Many men remain unaware of their BRCA status, access to genetic testing is variable, and there are no established pathways for systematically identifying eligible individuals. The real‐world impact of this recommendation may therefore be limited.

The most notable finding of this survey was the divergence in views relating to targeted screening in Black men and those with a family history of prostate cancer. Although Black men have a higher incidence of prostate cancer, it remains uncertain whether screening this group would reduce mortality.^6^, ^7^ In addition, men of Black ethnicity have been under‐represented in major prostate cancer screening trials, limiting the generalisability of existing evidence. This uncertainty is likely reflected in the lower confidence in the evidence reported by respondents and highlights the need for contemporary data from ongoing studies such as TRANSFORM.^8^ The NSC modelling suggests that screening men with a family history (approximately one‐third of all men) would result in similar levels of overdiagnosis to whole population screening. Despite this, two thirds of respondents did not agree with the recommendation. In practice, family history would be exceedingly difficult to operationalise, as it is unreliably recorded in electronic health records with no standardised definition to stratify risk, limiting the feasibility of targeted screening in this group. Our survey included a high proportion of urologists involved in diagnosing and treating prostate cancer but was limited by the low overall response rate.

In conclusion, this national survey highlights a clear divide between the views of urologists and most recent screening policy. Whilst clinicians broadly support recommendations against whole population screening and in favour of screening those with known BRCA1/2 mutation, there is a clear lack of agreement with recommendations against screening in other higher‐risk subgroups. These findings suggest that many clinicians who guide men through the diagnostic and treatment pathway do not view this as a binary choice between screening and no screening, but a requirement for a more nuanced, risk‐stratified approach.

## AUTHOR CONTRIBUTIONS

NM and HUA conceived the study. NM designed and distributed the survey, analysed the data, and drafted the manuscript. RG contributed to study methodology, statistical interpretation, and critical revision of the manuscript. FR, EC, and VSP contributed to data collection, interpretation of the findings, and critical revision of the manuscript. HUA supervised the study and contributed to interpretation and critical revision of the manuscript. All authors reviewed and approved the final version of the manuscript.

## CONFLICT OF INTEREST STATEMENT

Hashim U. Ahmed and Rhian Gabe are co‐leads of the TRANSFORM prostate cancer screening trial. Nikhil Mayor, Francesca Rawlins, Emma Cullen and Varsha Sonigra‐Patel are co‐investigators for TRANSFORM. No financial conflicts of interest are declared.

## Data Availability

The data that support the findings of this study are available on request from the corresponding author. The data are not publicly available due to privacy or ethical restrictions.
